# The Ohio Dentists and the Goodyear Dental Vulcanite Company

**Published:** 1866-11

**Authors:** 


### THE OHIO DENTISTS AND THE GOODYEAR DENTAL VULCANITE COMPANY.

THE Ohio State Dental Society held a called meeting in Columbus, Nov. 1st, to consult in reference to the claims of the Goodyear Dental Vulcanite Company. Dentists present who were not members of the society, were invited to participate in the discussions and proceedings. After hearing the report and opinions of the attorney employed in the case, and after a very free exchange of opinion, it was unanimously agreed not to accede to the present demands of the Goodyear Dental Vulcanite Company.

